# Regulation of Mitochondrial Genome Inheritance by Autophagy and Ubiquitin-Proteasome System: Implications for Health, Fitness, and Fertility

**DOI:** 10.1155/2014/981867

**Published:** 2014-06-17

**Authors:** Won-Hee Song, John William Oman Ballard, Young-Joo Yi, Peter Sutovsky

**Affiliations:** ^1^Division of Animal Science, and Departments of Obstetrics, Gynecology and Women's Health, University of Missouri, Columbia, MO 65211-5300, USA; ^2^School of Biotechnology and Biomolecular Sciences, University of New South Wales, Sydney, NSW 2052, Australia; ^3^Division of Biotechnology, College of Environmental & Bioresource Sciences, Chonbuk National University, Iksan-si, Jeonbuk 570-752, Republic of Korea

## Abstract

Mitochondria, the energy-generating organelles, play a role in numerous cellular functions including adenosine triphosphate (ATP) production, cellular homeostasis, and apoptosis. Maternal inheritance of mitochondria and mitochondrial DNA (mtDNA) is universally observed in humans and most animals. In general, high levels of mitochondrial heteroplasmy might contribute to a detrimental effect on fitness and disease resistance. Therefore, a disposal of the sperm-derived mitochondria inside fertilized oocytes assures normal preimplantation embryo development. Here we summarize the current research and knowledge concerning the role of autophagic pathway and ubiquitin-proteasome-dependent proteolysis in sperm mitophagy in mammals, including humans. Current data indicate that sperm mitophagy inside the fertilized oocyte could occur along multiple degradation routes converging on autophagic clearance of paternal mitochondria. The influence of assisted reproductive therapies (ART) such as intracytoplasmic sperm injection (ICSI), mitochondrial replacement (MR), and assisted fertilization of oocytes from patients of advanced reproductive age on mitochondrial function, inheritance, and fitness and for the development and health of ART babies will be of particular interest to clinical audiences. Altogether, the study of sperm mitophagy after fertilization has implications in the timing of evolution and developmental and reproductive biology and in human health, fitness, and management of mitochondrial disease.

## 1. General Introduction to the Origin of Mitochondria and Unique Features of the Mitochondrial Genome

### 1.1. Mitochondria and Their Origin

Mitochondria exist in almost all eukaryotic cells. They are semiautonomous, having their own genome and their transcriptional and protein synthesizing machinery [1]. Mitochondria play an important role in numerous cellular functions including calcium signaling, programmed cell death (apoptosis), cellular aging, and energy generation. They generate cellular adenosine triphosphate (ATP) and control the machinery for cellular differentiation, cell death, and cell cycle [2].

The origin of mitochondria from a bacterial symbiont has been widely accepted. The most frequently cited hypothesis to explain the origin of mitochondria is the endosymbiosis theory proposed by Margulis [3]; it states that mitochondria descended from free-living eubacteria, which we know now, have their own DNA and functioning protein synthesis system. The Margulis theory, harshly criticized at the time, postulated that the nucleus came from an archaebacterium and the symbiotic relationship began with an eubacterial progenitor of the modern mitochondria [4].

The origin of the mitochondrial lineages is associated with increasing oxygen levels in the atmosphere. The consumption of oxygen by metabolism produces energy in the form of ATP. It was previously believed that the symbiosis between the host and the endosymbiont was based on the endosymbiont producing ATP and the host cell was using carbohydrates exchanged by ATP. However, anaerobic ATP-producing mitochondria are present in unicellular protists and nematodes. They depend on NO_2_^−^ and NO_3_^−^ rather than O_2_. Hydrogenosomes, which are another type of anaerobic ATP-producing organelle, are linked to H_2_ for ATP production, harboring O_2_-sensitive enzymes including pyruvate:ferredoxin oxidoreductase and hydrogenase [5, 6].

### 1.2. Genes Encoded by mtDNA and Their Function

In most eukaryotic cells, mitochondrial DNA (mtDNA) is composed of a circular, double-stranded DNA, where the inner circle represents the cytosine-rich light strand (L-strand) and the outer circle represents the guanine-rich heavy strand (H-strand). The mitochondrial genome in mammals encodes 37 genes: 13 protein coding, 22 mitochondrial tRNA coding, and two genes coding 12S and 16S rRNAs. The mtDNA contains a non-coding region, referred to as the D-loop in mammals or the A + T rich region in other organisms such as insects, in which the mitochondrial transcription promoter is situated. In addition, mtDNA replication also starts in the D-loop. The mitochondrial genetic code is different from nuclear DNA; AGA and AGG are read as stop codons; AUA and AUU are start codons in mitochondrial genes [7]. The major function of genes encoded by mtDNA is the production of core proteins essential for oxidative phosphorylation.

Oxidative phosphorylation in mitochondria is the metabolic pathway in which ATP is generated from electron transport chain located in the mitochondrial inner membrane space [8]. There are five complexes of the electron transport chain: respiratory complex I (NADH: ubiquinone oxidoreductase or NADH dehydrogenase, encoded by* Nad* genes), complex II (succinate: ubiquinone oxidoreductase), complex III (ubiquinol: cytochrome c oxidoreductase), complex IV (cytochrome c oxidase), and complex V (ATP synthase) [9]. Complex I consists of 45 subunits including 14 core subunits and 31 accessory subunits; 7 core subunits are encoded by mDNA and 7 core subunits encoded by nuclear DNA in bovine [10]. Complex II is composed entirely of nDNA-encoded subunits. Complex III consists of one mtDNA-encoded subunit and 11 nDNA-encoded subunits [7]. Complex IV contains three mtDNA-encoded respiratory chain subunits while the remaining 11 are nuclear DNA [11]. In complex V, only 2 out of 19 subunits are coded by mtDNA [12]. In human mtDNA, complex I discards electrons by NADH, whereas complex II gathers electrons. The electrons are moved to coenzyme Q by both complexes and flow though complex III, then to complex IV to produce water. The electron chemical ingredient that is generated by electron transport chain is utilized for complex V to produce the energy source, ATP [13].

### 1.3. mtDNA Differs from Nuclear DNA

Nuclear DNA is organized into bead-like structure, the nucleosomes that contain DNA wrapped around core histones and are cross-linked with linker histone H1. The nucleosome consists of two sets of four different histone proteins including H2A, H2B, H3, and H4, and 170 base pairs of DNA, forming a coiled structure. Histones have the basic DNA-binding proteins that control multiple aspects of DNA function [14]. In contrast to nuclear DNA, mtDNA is not afforded protection and structural organization conveyed by histones. The rRNA and tRNA coding portion of mtDNA chain is packaged into histone-free nucleoids [15] by proteins, most abundantly the mitochondrial transcription factor A (TFAM). The TFAM acts as a protective and regulatory packaging of mtDNA, protecting it from oxidative damage and promoting transcriptional initiation [16]. Interestingly, our previous study on porcine spermiogenesis has provided evidence that sperm TFAM protein is ubiquitinated and segregated from the mitochondria of elongated spermatid to nonmitochondrial region, the principal tail piece of fully differentiated boar spermatozoa. The ubiquitination of TFAM protein in spermatozoa could mediate the recognition of paternal mitochondria by ooplasmic protein/organelle degradation machinery and preclude the transcription of paternal mitochondrial genes in the fertilized oocyte [17].

Nuclear DNA also differs from mtDNA in its structure. Cytological examination reveals that the chromosomal DNA found in the nucleus of most eukaryotes is linear. Consequently, the size and form of chromosomal DNA are considered invariant across generations and tissues [18]. In contrast, mtDNA is circular in most eukaryotes. An exception to this rule is* Schizosaccharomyces pombe*, in which 1% or less of mtDNA is circular and the majority of mtDNA molecules exist in linear and branched forms [19].

Another characteristic differentiating mtDNA from chromosomal DNA is its stability. Nuclear DNA is stable, whereas mtDNA displays significant instability. Induced DNA damage can negatively affect transcriptional regulation, as well as causing mutations due to the change of hydrogen bonding site, such as thymine glycol and 2-hydroxyadenine. Oxidative damage to DNA is the major source of mutation in eukaryotes. The oxidative DNA damage is higher in mtDNA than in nuclear DNA. Mitochondrial DNA is relatively more vulnerable to damage and displays mutations at a 10- to 50-fold higher rate compared to the nuclear genome [20, 21]. In* Drosophila*, the overall mtDNA mutation rate is 10 times higher than that of nuclear DNA [22]. The high rate of mtDNA mutation is posited to be due to its proximity to sites of reactive oxygen species (ROS) production, the lack of protective histones, and limited DNA repair ability; there are, however, two recognized mtDNA repair systems in mammalian cells, the nucleotide excision repair pathway and the base excision repair pathway [23]. This high rate of mutation results in the somatic accumulation of mutations as an organism ages [24].

Mitochondria are now known to be far more dynamic than was thought few years ago, both in their number and localization within a cell, and with regard to copy number. Typically there is a single copy of each nuclear chromosome in each cell. In contrast, there is substantial variability in cellular mtDNA copy number between different organisms and cell types, typically ranging from less than one hundred to several thousands of mtDNA copies per cell [7]. The copy number of mtDNA also changes with exercise [25]. Furthermore, mitochondrial function is linked with the expression of genes encoded by nuclear DNA; the nuclear genome responds to mitochondrial dysfunction by changes in nuclear gene expression, including but not limited to genes in ubiquitin-proteasome system [26] which, as will be discussed later, contribute to the regulation of mitochondrial inheritance. At the subcellular/organelle level, mitochondria respond to physiological and pathological stimuli by changes in the rate of fusion/fission, shape, and subcellular localization and, when damaged or outlived, are subject to selective removal [27].

Other unique features of mtDNA include the lack or rare occurrence of recombination [28, 29] and the predominantly maternal inheritance in most animal taxa [15]. This uniparental pattern of mtDNA inheritance is called clonal or maternal inheritance. Although maternal inheritance of mtDNA is predominant in eukaryotes, there are different variations of it and we discuss these in the next section.

## 2. Mechanism of mtDNA Inheritance in Animal Sexual Reproduction

### 2.1. Parental Modes of mtDNA Inheritance

The inheritance of mitochondrial genome differs from nuclear genomes with biparental inheritance, as the inheritance of mtDNA does not follow a Mendelian pattern. Consequently, mtDNA is strictly inherited from mitochondria of the mother's oocyte in most animals. Some organisms inherit only maternal or paternal mitochondrial genes while others get them from both parents. For example, MtDNA is biparentally inherited in the yeasts* Saccharomyces cerevisiae* and the fission yeast* Schizosaccharomyces* [30].

Over the years, scientists have studied mtDNA inheritance patterns in a variety of organisms. Paternal inheritance of mtDNA occurs in mussels [31], including the families Mytilidae (sea mussels) and Unionidae (fresh water mussels), which have two different types of mtDNA, the F type and the M type. The F type mtDNA is transmitted to the maternal lineage and the M type mtDNA through paternal lineage. This inheritance mechanism has been called doubly uniparental inheritance (DUI). The inheritance pattern of mitochondrial genome is more variable in the interspecific crosses between two species of mouse,* Mus musculus* and* M*.* spretus*, used to study the mechanism of mitochondrial inheritance in mammals [32]. Due to the failure of sperm mitophagy after fertilization, molecules of paternal mtDNA were detected in hybrid* M. musculus* ×* M*.* spretus* embryos by nested PCR method. Paternal, sperm-derived contingent of mtDNA was estimated at 1–4 molecules per 100,000 maternal, oocyte derived mtDNA molecules. Paternal lineage of mtDNA was also found in an interspecific cross between* Drosophila simulans* and* Drosophila mauritiana* [33], and in the heteroplasmic* D. simulans* lines [34]. It appears that sperm mitochondria are not recognizable to ooplasmic mitophagy machinery in the interspecific crosses, and occasionally, or in some species and lineages regularly, a small amount of paternal mtDNA escapes degradation in the oocyte cytoplasm of interspecific crosses [35].

Although paternal inheritance occurs in mussels and interspecific crosses of* Drosophila* and mouse, paternal mitochondria and their mtDNA cargo is selectively eliminated after fertilization in most animals. Therefore, maternal inheritance of mtDNA is regarded as the major rule of mtDNA transmission in animals and humans.

### 2.2. Genetic Bottleneck during Gametogenesis

With the exception of interspecific crosses in animals and rare leakage of paternal mtDNA reported in humans [36], the mammalian mitochondrial genome is maternally inherited. However, due to the mutation-prone nature of mtDNA, a mixture of wild-type and mutated mtDNA can be transmitted from mother to progeny, resulting in a condition of two mitochondrial genomes coexisting in the same individual, termed heteroplasmy. High levels of heteroplasmy from maternally transmitted mtDNA mutations are thought to cause mitochondrial disease and have abnormal phenotypes [37, 38]. The mitochondrial bottleneck theory explains the change in mtDNA mutant levels, which indicates a dramatic reduction in mtDNA during embryonic development. The bottleneck is present between the development of primordial germ cell and primary oocyte. The number of replicating mtDNA is increased in the primordial oocyte. Early on, the mitochondrial bottleneck was considered to have a negative effect, causing mitochondrial disorders in progeny. However, it now appears that the mtDNA bottleneck prevents accumulation of adverse mtDNA mutation in the maternal germ line, thus protecting the species by reducing the transmission of mutated mitochondrial genes [39, 40]. This could also affect the male germ line, if mtDNA mutations cause altered mitochondrial function and reduced sperm motility once the affected male germ cells differentiate into spermatozoa [41]. However, due to sperm mitophagy after fertilization, the paternal mtDNA mutations would not be transmitted to offspring.

The mitochondrial bottleneck theory proposes a segregation mechanism of both mutant and wild-type mtDNA. However, the precise explanation of this segregation is still missing. There are three possible mechanisms of the mtDNA bottleneck. First, variation in heteroplasmy is caused by the unequal segregation of mutant and wild-type mtDNA during cell division [37]; second, variation in heteroplasmy is caused by the unequal segregation of homoplasmic nucleoids from multiple mtDNAs during cell division [42]; third, variation in heteroplasmy is caused by the mitochondrial genomes selected for replication [39, 43].

In mice, fertilized oocytes develop into blastocysts by day 4.5 following conception (d.p.c), which is called preimplantation development. The mtDNA copy number remains constant during the preimplantation period [37]. Cree et al. [37] showed that 70% of the heteroplasmy variance was seen at 7.5 d.p.c, which leads to the physical restriction of mtDNA content in early post implantation development of heteroplasmic mice. The remaining 30% were produced during increased proliferation of mtDNA in the expanding germ line. Consequently, Cree and colleagues proposed that the mitochondrial bottleneck is due to the unequal segregation of mtDNA in early postimplantation development [37]. However, Cao et al. [42] concluded that the mitochondrial bottleneck is not due to the reduction of mtDNA copy number but is caused by the segregation of multiple homoplasmic copies of mtDNA. Despite several hypotheses being proposed, the exact mechanism of the mitochondrial bottleneck is still unknown. In essence, the mitochondrial bottleneck could remove mutations in mtDNA from maternal lineage. This process will then prevent mitochondrial disease in subsequent generations.

### 2.3. Theories Explaining Clonal mtDNA Inheritance

The fate of paternal mitochondria after fertilization has been a controversial issue in past decades. Early studies presumed that the paternal mitochondria participated in the early embryonic development, while others erroneously thought that the sperm tail with paternal mitochondria was jettisoned before it entered the oocyte at fertilization. As early as 1965, Szollosi [44] reported for the first time that the sperm mitochondria sheath is disassembled by the early eight-cell stage of preimplantation development in the rat embryo.

Although there is no longer doubt that sperm mitochondria enter the oocyte at fertilization, misconceptions about the fate and contribution of paternal mitochondria during preembryo development were still perpetuated in 1980s and 1990s [45]. In early studies, Wilson et al. [46] suggested that vertebrate spermatozoa carry mitochondria and mtDNA in the sperm tail midpiece, but sperm mitochondria do not enter oocyte cytoplasm, or if they do, few survive past one-cell stage [46]. More recently, Lewin incorrectly suggested in a cartoon that the sperm tail including mitochondria is discarded before entering the oocyte at the time of fertilization [47]. Dawkins also reported that mitochondria are inherited from the maternal lineage because the tiny size of a spermatozoon is insufficient to support its own mitochondria [48]. The above hypotheses are not supported with current knowledge showing that sperm mitochondria do enter the mammalian oocyte at fertilization and are actively degraded inside oocyte cytoplasm after fertilization.

Four possible mechanisms should be considered for the lack of paternal mitochondrial gene transmission. The first is a simple dilution effect. A single spermatozoon contains approximately one thousand times less mtDNA molecules than an oocyte [36]. Second, the mitochondrial bottleneck could amplify the dilution of minor paternal alleles during embryonic development [7]. Third, by the time it reaches the oocyte, the fertilizing spermatozoon could contain degraded mtDNA or no mtDNA at all, as recently proposed in* Drosophila* [49] and mouse [50]. Fourth, the active degradation process involving ubiquitination of paternal mitochondria followed by autophagy of the whole mitochondria or proteasomal degradation of extracted mitochondrial proteins acts as a signal for selective elimination inside the oocyte cytoplasm [51]. Evidence for the proteolytic mechanism of sperm mitochondrion elimination has been provided by studies in primate, ungulate, and rodent mammals. More recently, studies linking ubiquitin-proteasome system and autophagy during sperm mitophagy have been conducted in* Caenorhabditis elegans* [52, 53]. Different species seem to apply various mechanisms to prevent the transmission of paternal mitochondria, as will be discussed below in more detail.

### 2.4. What Is the Advantage of Clonal mtDNA Inheritance for Individual Fitness and Species Survival?

The transmission of mitochondria and mtDNA through the female germ line gives rise to male-female asymmetry, whereas the nuclear but not the mitochondrial genome is contributed equally by both parents. This uniparental inheritance may avoid lethal conflict between genomes [54]. Mitochondria produce ROS that could alter mtDNA integrity; ROS produced during cellular oxidative phosphorylation (OXPHOS) has the potential for causing mutagenic and cytotoxic effects [7]. Therefore, oxidative stress results in a high rate of mtDNA mutation, leading to accumulation of harmful mutated mtDNA known as Muller's Ratchet [55].

Approximately 500 different mtDNA mutations have been associated with degenerative human diseases, cancer, and aging [56–58]. A single pathogenic mtDNA mutant, Leber's hereditary optic neuropathy, is the first known maternally transmitted mitochondrial disorder. Age-related neurodegenerative disorders including Parkinson's and Alzheimer's disease are strongly associated with impaired mitochondrial function [59].

The asymmetric inheritance of mtDNA has an adverse effect on male fitness and male fertility. MtDNA mutations that affect males do not respond to natural selection, though some evidence of sexual selection based on mtDNA sequence was reported in humans [60]. Highly detrimental mutations in mitochondrial genomes are eliminated during female germline development whereas mildly deleterious mutations may be transmitted to the next generation though the female germline [61]. Natural selection will, however, put more pressure on the elimination of female-specific slightly deleterious mutations than male-specific ones because mitochondria are maternally inherited. This selection asymmetry in mtDNA has been described as the mother's curse effect [55]. There is, however, evidence to suggest that specific mtDNA mutations can cause physiological tradeoffs through cellular signalling, which can result in evolutionary advantage in some circumstances [62]. Collectively, these studies illustrate the complexity of mitochondrial inheritance.

Poor male fitness and sperm dysfunction may result from mtDNA mutations that cause a decrease of OXPHOS efficiency. Spermatozoa that possess few mitochondria demand high bioenergetic efficiency for motility, whereas oocytes have low energetic requirements yet possess many times more mitochondria per cell [55]. Therefore, female fertility may be unaffected by a mutation that is detrimental to male fertility. However damaging it may be to male fertility, low levels of deleterious mutated mtDNA will not affect male's fitness until the levels of mutated mtDNA reach a mutation specific threshold level [55, 63].

### 2.5. Assisted Reproduction and Mitochondrial Fitness

In an effort to alleviate mitochondrial disease, recent research offers experimental clinical techniques aimed at preventing the transmission of mutant mtDNA [64, 65]. In patients with mitochondrial disease, the mutant mtDNA is either homoplasmic (all mtDNA copies are mutated) or heteroplasmic (both mutant and wild type mtDNA found in the same individual) [66]. The disease phenotypes are only present in patients with above-disease threshold level of mutant mtDNA. One approach to reduce the harmful effect of mutant mtDNA in human embryos created by ART is to transfer the nuclear genome from the zygote with abnormal mitochondria to the recipient zygote/ooplast with healthy mitochondria [64]. Such a pronuclear transfer resulted in normal development to blastocyst, regardless of whether one or two pronuclei were transferred. The pronuclear transfer technique was optimized to minimize the size of the karyoplast with a small amount of cytoplasm, which represents the mtDNA carryover of donor zygote [67]. It remains to be determined what effect, if any, the potential incompatibility between karyoplast nuclear genome and donor cytoplast mitochondrial genome will have on embryo development. A variation on this technique to prevent transmission of mutant maternal mtDNA is to transplant the metaphase II spindles from unfertilized oocytes containing abnormal mitochondria to those of healthy recipient oocytes, as studied in nonhuman primates [65]. The technique involving the metaphase II spindle transfer has a potential for reducing the level of the carryover mtDNA. Therefore, both the pronuclear and spindle transfer techniques might have the potential for reducing the transmission of mtDNA diseases.

An application of mitochondrial therapy that is particularly relevant to human ART is mitochondrial replacement (MR) therapy. It is well established that oocytes of ART patients of advanced reproductive age (past 35 years of age) have significantly reduced developmental potential, which may be contributed by suboptimal mitochondrial function. Consequently, successful attempts were made early in the century to rejuvenate aged oocytes by infusion of cytoplasm/mitochondria harvested from oocytes provided by donors of prime reproductive age [68]. It was found that this cytoplasmic transplantation generates mtDNA heteroplasmy from donor to offspring in amniocytes, placenta, and fetal cord blood. These attempts were stopped and the ooplasmic transplantation/MT procedure was banned in US and abroad after a report of high incidence of birth defects/developmental anomalies in MR babies [69]. However, the technique has been reintroduced recently in UK, sparking concerns about prenatal development, and postnatal health and fitness of MR children. In particular, numerous examples of detrimental effects of MR/experimentally induced heteroplasmy on health and fitness of MR offspring generated by genetic crossing techniques or by organelle transfer exists in animal models ranging from insects to nonhuman primates [70]. Consequently, some maintain that the reintroduction MR therapy in ART clinics appears premature in absence of extensive animal model testing and safeguarding of human treatments.

## 3. Maternal Inheritance of mtDNA in Yeast, Nematodes, and Mammals

### 3.1. Yeast Mitochondria

A yeast cell consists of a single mitochondrial network that exhibits the tubular-reticular structure. Mitochondrial inheritance and dynamics in yeast are maintained by distinct mechanisms: the fission and fusion of mitochondria, the mitochondrial segregation from mother to daughter, and the maintenance of mitochondria [71]. Mitochondrial inheritance is closely synchronized with the cell cycle. Mitochondria align along the mother-bud axis in G1 phase and linear movement of mitochondria from the mother to the bud site occurs during S phase. Mitochondria become immobilized in the bud tip during G2 phase and are eventually released from bud and equally divided between the mother and daughter cell during M phase [72].

Mitochondria in yeast undergo fusion and fission during cell growth, mating, and sporulation. Fusion occurs between the two sides of mitochondrial tubule. Fission occurs along the length of mitochondrial tubules [71].* Fzo1*, a homologue of the* Drosophila fuzzy onions*, is required for mitochondria fusion in yeast. Fzol protein localizes to the outer mitochondrial membrane.* Fzo1* mutants display mitochondrial fragmentation and loss of mtDNA [73, 74]. The dynamin Dnm1p, a GTP-binding protein, is located in mitochondria at division sites. Dnm1p is required for mitochondrial fission and essential for normal mitochondrial morphology in yeast. The* Fzo1* and* Dnm1p* double-mutant lack fusion and fission activities. Therefore, normal mitochondrial morphology is controlled by the balance between the expression of* Fzo1* and* Dnm1p* [35, 74].

Mitochondrial transmission from mother to bud progresses along a cytoskeletal track composed of actin patches and actin cables. Mitochondrial movement thus depends on the actin cytoskeleton. The depolarization of actin patches and cables results in the accumulation of mitochondrial particles and loss of mitochondrial movement and abnormal mitochondrial distribution [72, 75]. Mmm1p, Mdm10p, and Mdm12p proteins of the outer mitochondrial membrane are required for segregation of mitochondria to the daughter cell, mtDNA maintenance, and normal mitochondrial morphology [71, 76].

### 3.2. Nematode Gamete Mitochondria

The nematode* C. elegans* is a self-fertilizing hermaphrodite, having a reproductive organ producing first spermatozoa then oocytes. Self-fertilized embryos proceed through development, hatch, and reach larval stages (L1–L4) [77]. In* C. elegans*, mtDNA inheritance and maintenance for heteroplasmy have been reported [78]. The* uaDf*5 mutation is a 3.1 kb deletion that removes a total of 11 genes; it is maternally inherited and has been transmitted for many generations [78]. Because the* uaDf*5 mutation is not viable as homoplasmic, it is maintained at ~60% of the mtDNA contents in a stable heteroplasmic condition throughout development. The possible reason for the maintenance of stable heteroplasmy may be that the short, deleted mtDNA molecules have a replicative advantage [79]. Smaller chromosomes could replicate faster over wild-type containing larger chromosomes. However, there is no replicative advantage in the* uaDf*5 mtDNA deletion due to the stable level of* uaDf*5 through the development [77].

Observations in* C. elegans* suggest that deletions induced by the removal of the large portion of mitochondrial chromosome result in mtDNA mutation that has severe effects on the carrier's fitness [80]. The experimentally induced mtDNA deletion decreased sperm performance and fitness. It shows that heteroplasmic spermatozoa carrying* uaDf*5 deletion of mtDNA crawled more slowly than spermatozoa with wild-type mtDNA, although the sperm fertility was unaffected. The slower rate of egg-laying and shortened life span is also caused by mtDNA* uaDf*5 deletion [80].

### 3.3. Mammalian Sperm Mitochondria

In mammals, mtDNA is typically maternally inherited. Sperm mitochondria are eliminated in the early preimplantation embryo. Although this uniparental inheritance appears to be prevalent in mammals, the underlying developmental mechanisms and timing of sperm mitophagy may vary to some extent among species/taxa. The paternal, sperm borne mitochondria enter the oocyte cytoplasm upon fertilization and are temporally present at the onset of embryonic development. The sperm-contributed mitochondria and mtDNA are degraded and no longer detectable by the time of implantation [45].

Early studies of the elimination of sperm-derived mitochondria in mammals were conducted in mouse, rat, hamster, cow, and pig embryos. Sperm mitochondria are still detected at the two-cell stage in the mouse, and the four-cell stage in the rat and bovine embryo, although some other sperm flagellar structures such as the fibrous sheath disappear before the first embryo cleavage [81]. In the hamster zygote, multivesicular bodies accumulate around the sperm tail and fuse with the mitochondria before their degradation at the two-cell stage [82]. In the bovine embryo, disposal of the sperm mitochondrial sheath is completed in the four-eight cell embryo [83]. Sperm mitochondria in the pig zygote are degraded earlier than the bovine zygote and are undetectable after the first embryo cleavage [84].

Multiple modes of mtDNA inheritance have been described in mammals propagated through interspecies crossing or assisted reproduction. The sperm mtDNA transmission has been observed in various interspecies crosses including sheep interspecies hybrids [85], cloned nonhuman primate [86], and crosses of domestic and wild mice [87]. Kaneda et al. [87] detected paternally inherited mtDNA in* M. musculus* and* M. spretus* interspecies hybrids, whereas sperm mtDNA was eliminated at the early pronucleus stage in one-cell embryos of intraspecific crosses. In this study, interspecific crosses transmitted sperm-contributed mtDNA throughout early embryonic development to the neonate. Additionally, sperm mitochondria from a congenic strain, which derived mtDNA from* M. spretus* and mitochondrial proteins from* M. musculus* nuclear genes, were eliminated by* M. musculus* oocytes presented as intraspecific crosses. This observation suggested that paternal mitochondrial membrane proteins rather than paternal mtDNA itself were recognized by the ooplasmic machinery that seeks and destroys sperm mitochondria. Studies by Gyllensten et al. [32] also found that sperm mtDNA is present in the first generation offspring but not inherited in by the subsequent generations of mouse hybrids between* M. musculus* C57BL/6J strain and* M. spretus*. It can be concluded that the elimination of sperm mtDNA is a species-specific mechanism that is circumnavigated by mouse interspecific hybrids.

Somatic cell nuclear transfer (SCNT) is inherently conducive to heteroplasmy because donor somatic cell mitochondria likely lack the protein marks that would make them recognizable to the ooplast as being foreign mitochondria. Besides the mismatch between donor and recipient mitochondrial genomes, abnormal interactions of mitochondrial proteins encoded by mtDNA from a recipient oocyte with a donor cell's mitochondrial proteins encoded by donor-nuclear DNA could contribute to reduced fitness of embryos reconstructed by SCNT. In previous studies of bovine [88], murine [89], and porcine [90] SCNT offspring, heteroplasmy derived from the transmission of donor cell mtDNA was observed at varied ratios of donor-to-recipient mtDNA. A relatively small level of donor cell mtDNA was detected in the naturally conceived offspring of cloned pigs, which is in accordance with natural segregation of donor mtDNA leading to the concept of a genetic bottleneck in cloned germ line [90]. The ratio of donor mtDNA in cloned offspring reveals tissue-specific distribution in mouse brain, liver, and tail [89]. Interspecies somatic cell nuclear transfer (iSCNT) has been applied to various species, including gaur/bovine [91]. In gaur/bovine iSCNT embryos, the donor mtDNA from fibroblast was detectable at all stages of preimplantation development with varying degree of heteroplasmy among individual cloned embryos [91]. High levels of mtDNA heteroplasmy might contribute to lesser efficiency of iSCNT compared to intraspecific SCNT.

## 4. Sperm Mitophagy in the Mammalian Zygote Is Mediated by Ubiquitin-Proteasome System

How could sperm-borne mitochondria be selectively degraded after fertilization in mammals without the mitophagy mechanism attacking the oocyte's own mitochondria? Early work in bovine, rhesus monkey, and murine zygotes showed that once they enter the oocyte cytoplasm, the sperm mitochondria are tagged with ubiquitin, which is thought to flag the proteins and possibly organelles for degradation by the ubiquitin-proteasome system (UPS) [92]. In bovine* in vitro* embryo culture, paternal mitochondria are eliminated between 4- and 8-cell stages. Interestingly, sperm mitochondria in interspecific bovine cross embryos were not ubiquitinated and still present at the eight-cell stage [92]. Such an observation agrees with the persistence of paternal mtDNA in the interspecific mouse crosses [93], suggesting that the mechanism for selective sperm mitophagy has a species-specific element.

The UPS is an essential, tightly regulated route for substrate-specific protein degradation in eukaryotes. Consequently, UPS is a good candidate for selective degradation of paternal mitochondrial proteins after fertilization [94]. Ubiquitin is a highly conserved small proproteolytic chaperone protein present in the cell cytoplasm and nucleus and, in some instances, in the extracellular space and on the cell surface [95]. Protein ubiquitination signifies the covalent ligation of one or more ubiquitin molecules to substrate proteins through a sequential action of at least three enzyme activities: a ubiquitin-activating enzyme E1, a ubiquitin-conjugating enzyme E2, and a substrate-specific ubiquitin ligase E3 [96]. Upon the ligation of one ubiquitin molecule to substrate's internal Lys residue (monoubiquitination), additional ubiquitin molecules can attach to one of the Lys-residues of that substrate-bound monoubiquitin in a tandem fashion, again through linkage to one of its seven internal Lys-residues (K6, K11, K27, K29, K33, K48, and K63), to form multiubiquitin chains [97].

Protein tagging by polyubiquitin chains culminates in recognition and proteolysis by the 26S proteasome, a multisubunit protease holoenzyme. The 26S proteasome is typically composed of a 20S proteasomal core particle, a complex of four concentric rings forming a hollow barrel-shaped structure, and a cap-like structure of the 19S proteasomal regulatory complexes on one or both ends of the 20S core. The 19S complex recognizes, engages, and removes the multiubiqitin chain on the ubiquitinated substrate protein, which is then primed and translocated to the 20S core for degradation by three resident proteases (20S core subunits PSMB5, PSMB6, and PSMB7). The substrate protein is broken down into small peptides of 3–20 amino acids, released from the proteasome and degraded to individual amino acids by cytosolic endopeptidases [97, 98].

During mammalian spermatogenesis, mitochondrial ubiquitination is detected at the secondary spermatocyte phase, followed by the round spermatid, and finally in fully differentiated testicular spermatozoa of the bull [94]. After incorporation into the oocyte cytoplasm during fertilization, ubiquitinated sperm mitochondria are detectable in the oocyte cytoplasm whereupon the intensity of ubiquitin binding appears to increase, as detected by immunofluorescence imaging [51]. In porcine zygotes, the degradation of the sperm mitochondrial sheath was delayed by the specific, reversible proteasomal inhibitor MG132 and resumed once the inhibitor was washed off [84]. Paternal mitochondria in porcine zygotes appear to be degraded prior to the first embryo cleavage following* in vitro* fertilization. Lactacystin, an irreversible proteasomal inhibitor, also prevents sperm mitophagy in the porcine zygote. Such data indicate that the elimination of ubiquitinated proteins can be controlled by proteasomal activity, which, as we will discuss below, is linked to autophagic pathway for organelle degradation. The proteasomal inhibitors also block the penetration of the mammalian egg coat, zona pellucida (ZP), which suggests the role of sperm proteasome during the sperm-ZP interactions [84]. Consequently, proteasomal inhibitors had to be added to the fertilization medium after sperm-zona penetration was completed, in order to assess their effect on sperm mitophagy.

## 5. The Role of Autophagic Pathway in the Sperm Mitochondrion Degradation

Following an early indication of lysosomes contributing to sperm mitophagy in the bovine embryo [94], recent studies in* C. elegans* support the hypothesis that the ubiquitin and lysosome-dependent autophagic pathway actively participate in sperm mitophagy after fertilization. Autophagy, from the Greek word meaning “to eat oneself,” is conserved in all eukaryotes. Various macromolecules and whole organelles are delivered from the cytoplasm to lysosomes for degradation. In the process of autophagy, protein-aggregates, defective cellular structures, and damaged organelles are engulfed by double-membrane vesicles called autophagosomes. These vesicles with cargo destined for degradation are targeted to the lysosome, the major digestive organelle in the cell. After approaching the lysosomes, autophagosome-lysosome fusion occurs to form autolysosomes. Eventually, degradation takes place through the mediation of lysosomal hydrolase enzymes [99].

Three recent studies in* C. elegans* implicate lysosome and autophagy processes in the degradation of paternal mitochondria in the early embryo [52, 53, 100]. The degradation of sperm borne mitochondria is associated with the lysosome that is a digestive organelle containing hydrolytic enzymes. Treatment with NH_4_CI, a lysosome inhibitor, delays paternal mitochondrial elimination after fertilization. The participation of lysosome pathway implicates the involvement of autophagy pathway as an upstream process of the lysosomal degradation [100]. The* C. elegans* sperm mitochondria are near the ER/Golgi derived membranous organelle (MO), which is essential for sperm motility. Fertilization in* C. elegans* triggers a selective autophagic response. Sperm mitochondria and MOs in* C. elegans* enter the oocytes upon fertilization, and the paternal mitochondria disappear by the 16-cell stage of the embryonic development [52, 53]. Autophagosomal membrane proteins LGG-1 and LGG-2 (homologs of mammalian GABARAP and LC3, resp., Table 1) accumulate around both paternal mitochondria and MOs in the oocyte cytoplasm. Furthermore, paternal mitochondria persist in the late stage embryos past 16 cells in the LGG-1 knockout worms. It appears that the sperm-derived mitochondria are engulfed by the autophagosomes and eliminated by autophagic pathway in the nematode embryo. Notably, sperm-derived MOs are tagged with ubiquitin in the oocyte cytoplasm and are subsequently recognized by the autophagosome for autophagic degradation [52, 53]. Although paternal mitochondria are tagged with ubiquitin during spermatogenesis and after fertilization in mammals, paternal mitochondria do not appear to be ubiquitinated in* C. elegans*.

Interestingly, the comparative experiments in* C. elegans* [52, 53] suggest that sperm mitochondrion autophagy is also conserved in mammals. The autophagy related proteins, such as LC3, GABARAP, and p62 (Table 1), were detected around the midpiece of the fertilizing spermatozoa inside mouse embryos. Autophagy is triggered when mouse spermatozoa enter the oocyte after fertilization and may have functions other than sperm mitophagy since it appears to be required for mouse embryo survival; the* Atg5−/−* mouse embryos die by the eight-cell stage [101].

The accumulation of autophagosomal markers in the vicinity of ubiquitinated sperm mitochondria in* C. elegans* and mouse embryos reflects evolutionary conservation of the proteolytic mechanism for the elimination of paternal mitochondria after fertilization. Such findings also hint at the synergy between autophagy and UPS during sperm mitophagy inside the fertilized oocyte/zygote.

Recent study of mouse embryos challenged the role of autophagic pathway in murine mitochondrial inheritance [50]. Oocytes of transgenic female mice expressing the green fluorescent protein (GFP) tagged autophagosome protein LC3 were fertilized with spermatozoa of a transgenic strain expressing red fluorescent protein (RFP) in mitochondria. Authors found that red fluorescent structures purported to be sperm mitochondria remained detectable up to morula stage but the RFP fluorescence did not colocalize with GFP-LC3 or with LysoTracker labeled lysosomes, leading them to conclude that autophagic pathway is not required for the elimination of paternal mitochondria after fertilization. Lack of LC3 association with sperm mitochondria beyond early stages of embryo development agrees with our preliminary studies in porcine model (Figure 2) but by no means allows concluding that autophagy is not involved, since other branches of the autophagic pathway need to be considered. These authors also reportedly detected paternal mtDNA in a small proportion of mouse pups born after intraspecific crossing. As an alternative to postfertilization mitophagy, Luo et al. suggested that most mouse spermatozoa that reach the site of fertilization in the mouse oviduct lack mtDNA and speculated that male germ line mtDNA is degraded during spermiogenesis [50]. While intriguing, the proposed mechanism of passive elimination of paternal mtDNA fails short of explaining high incidence of paternal heteroplasmy in mouse interspecific crosses reported previously [32, 87]. It is also unclear whether red fluorescent structures detectable beyond the four-cell stage are intact sperm mitochondria or just remnant of mitochondrial membranes, and why it would be possible to detect paternal mtDNA in offspring if the sperm-mitochondrion derived RFP fluorescence was no longer detectable at blastocyst stage. Such data will likely be scrutinized from the point of view of sensitivity and specificity of paternal mtDNA detection in single spermatozoa, embryos, and offspring, as was the case recently in* Drosophila* [49, 102].

## 6. The Crosstalk between Autophagy and Ubiquitin-Proteasome System during Sperm Mitophagy

### 6.1. Possible Scenarios for the Synergy between Ubiquitin-Proteasome System and Autophagy during Sperm Mitophagy

**Figure 1:**
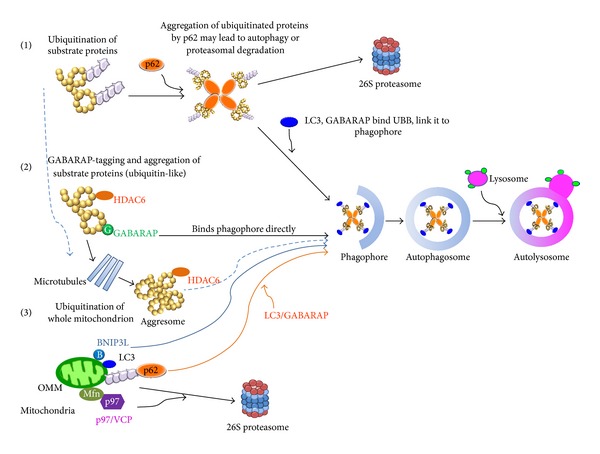
Diagram of candidate pathways leading to sperm mitophagy by autophagy and ubiquitin-proteasome system. Generally, the process of autophagy starts with the aggregation and ubiquitination of proteins or organelles that need to be recycled. Multiubiquitin chains on such aggregates are recognized by the ubiquitin-binding autophagy receptors and are brought to phagophore, a membranous organelle that eventually closes around the protein aggregate to form an autophagosome. In the finals step, autophagosome fuses with a lysosome that contains proteases able to degrade the protein cargo. In some branches of this pathway, protein aggregates or ubiquitinated proteins extracted from organelle membranes are targeted for degradation by the 26S proteasome, a multisubunit ubiquitin-specific protease. At least three previously characterized pathways could be involved in the degradation of sperm mitochondria inside a fertilized oocyte: (1) Autophagy-associated ubiquitin-receptor p62/SQSTM1 recognizes ubiquitinated cargo and interacts with autophagosome-binding ubiquitin-like proteins, such as LC3 or GABARAP; these autophagy receptors guide the protein cargo to phagophore; (2) ubiquitinated proteins of mitochondrial origin form aggresomes, the protein aggregates induced by the ubiquitin-binding adaptor protein HDAC6, which transport the ubiquitinated proteins towards degradation site, the phagophore, along microtubule tracks. (3) Protein dislocase p97/VCP extracts and presents the ubiquitinated mitochondrial membrane proteins to the 26S proteasome, the ubiquitin-dependent protease, without the involvement of phagophore.

Autophagy and the UPS are the two major cellular protein degradation pathways. Recent work has made it increasingly clear that ubiquitin-binding proteins/receptors in autophagy mediate interplay between the two systems. Hypothetically, at least three well characterized pathways involving autophagy and UPS could act in synergy during sperm mitophagy: (1) autophagy-associated ubiquitin-receptor p62/SQSTM1 recognizes ubiquitinated cargo and interacts with autophagosome-binding ubiquitin-like modifiers, such as LC3 or GABARAP; (2) ubiquitinated mitochondria or mitochondrial proteins may form aggresomes, the protein aggregates induced by the ubiquitin-binding adaptor protein HDAC6, which transport such ubiquitinated protein aggregates along microtubules; and (3) the protein dislocase p97/VCP has the ability to extract and present ubiquitinated mitochondrial membrane proteins to the 26S proteasome, the ubiquitin-dependent protease. All three pathways may converge at the time of the formation of autophagic vacuole, as shown in Figure 1. According to this scenario, the process of sperm mitophagy starts when the ubiquitinated sperm mitochondria or mitochondrial protein aggregates dock to preautophagic membrane of the phagophore, then become engulfed in autophagosome, and fuse with lysosome to complete organelle/aggregate proteolysis. Alternatively, some branches of the three pathways proposed above, and most notably the p97/VCP dependent pathway, could channel the dislocated ubiquitinated mitochondrial membrane proteins directly to the 26S proteasome. It should also be considered that UPS could regulate the autophagic branch of these pathways indirectly by controlling the turnover of autophagic ubiquitin receptor proteins.

### 6.2. Ubiquitin-Binding Proteins Serve as Autophagy Receptors

In the last decade, genetic screens identified approximately 30 autophagy-related (Atg) proteins in yeast that mediate autophagosome formation and cargo degradation in the autophagic body [103] (Table 1). Mammalian LC3 and GABARAP are located in the phagophore membrane and help attach ubiquitinated proteins to the autophagosome [104]. Both LC3 and GABARAP are members of the ubiquitin superfamily and facilitate selective degradation of ubiquitinated proteins [105]. The LC3 binds directly to the p62/SQSTM1 protein, which is found in inclusion bodies containing ubiquitinated protein aggregates and has a C-terminal ubiquitin-associated (UBA) domain that engages ubiquitinated proteins [106]. The SQSTM1 also contains a PB1 domain to facilitate self-oligomerization. A specific region of SQSTM1, known as the LC3 recognition sequence (LRS), is formed by the Asp337-Asp339 acidic cluster [106, 107]. The N-terminal *α*-helix surface of LC3 interacts with LRS and/or LIR (LC3-interacting region) of UBA domain. The SQSTM1-derived UBA domain can bind both Lys 48-linked and Lys 63-linked multiubiquitin chains [108].

Growing evidence suggests that the affinity of SQSTM1 to ubiquitinated proteins results in their subsequent transport to the autophagosome for degradation [109]. The SQSTM1, an autophagic adaptor, accumulates in damaged mitochondria and contributes to autophagic degradation [110]. The SQSTM1-deficiency inhibits the accumulation of LC-positive autophagosomes during amino acid starvation [109]. These findings strongly indicate that the ubiquitinated protein cargo is recognized by SQSTM1 and interacts with LC3 and/or GABARAP on the phagophore membrane. Preliminary evidence from our porcine mitophagy model [111] and comparative data from* C. elegans* and mouse [53] suggests that paternal mitochondria in mammals are degraded with the help of zygotic, oocyte-derived autophagy-associated ubiquitin-receptors.

### 6.3. Histone Deacetylase HDAC6 Links Transport Ubiquitinated Cargo along the Microtubule Cytoskeleton

The crosstalk between the autophagic pathway and the UPS may also utilize the histone deacetylase 6 (HDAC6), which has the ability to bind to misfolded ubiquitinated proteins on one side and to dynein-binding cytoskeletal adaptor proteins on the other [112]. The HDAC6 has an ubiquitin-binding zinc-finger, called BUZ domain, which facilitates ubiquitin-binding to HDAC6 and is necessary for aggresome formation and degradation [113]. HDAC6 does not possess a LIR motif but rather interacts directly with dynein motors, which transport the aggregated, misfolded proteins along microtubules to the microtubule-organizing center (MTOC) where aggresomes tend to accumulate. In mammalian fertilization, the zygotic centrosome organizes a microtubule sperm aster and is close to the sperm mitochondria since it is derived from the sperm-borne centriole [114]. Notably, failure of human assisted fertilization is associated with protein aggregation and proteasome accumulation around the sperm-derived zygotic centrosome [115]. Although the aggregated ubiquitinated proteins, also known as aggresomes, are not eliminated primarily by proteasomal proteolysis, they contain proteasomes; aggresome formation typically signals an increased need for protein recycling and sometimes indicates the saturation of UPS with undegraded ubiquitinated proteins, associated with cellular stress. HDAC6-deficient cells fail to concentrate misfolded proteins into aggresomes and will instead retain them as cytoplasmic aggregates [116]. These observations suggest that HDAC6 is a crucial mediator of aggresome formation and a candidate participant in zygotic sperm mitophagy.

Aggresomes are localized around the MTOC, and aggresome formation requires the microtubule network to transport polyubiquitinated misfolded proteins. Dysfunction of the MTOC or the dynein motor leads to disruption of aggresome formation [117]. This observation indicates that the HDAC6-engaged polyubiquitinated proteins bind to dynein motors and move toward the MTOC, delivering the aggresome for degradation. It has been proposed that the bulk-delivery of misfolded proteins in the form of aggresomes facilitates proteolysis via autophagic route, ending with fusion of the autophagosome and lysosome [116]. When the UPS is impaired, autophagy is accelerated to compensate for UPS dysfunction in HDAC6-dependent manner [118]. Importantly, recent studies suggest that HDAC6 mediates the clearance of damaged mitochondria. Ubiquitin ligase, Parkin, induces the ubiquitination of impaired/damaged mitochondria, leading to the recruitment of HDAC6. The target mitochondria are then cleared by HDAC6-dependent autophagy [119]. It can therefore be speculated that the sperm mitochondria could also be degraded by HDAC6 mediated selective autophagy.

Considering that a 26S proteasome can only degrade one protein molecule at a time (as opposed to a whole organelle or organelle membranes), it is prudent to ask how proteasomes could degrade a whole sperm mitochondrion inside a fertilized mammalian oocyte. To overcome this seeming paradox, one can focus on the ability of certain ubiquitin-binding protein dislocases to extract ubiquitinated proteins from the outer mitochondrial membrane and deliver them to the 26S proteasome. Best characterized among these “substrate-presenting,” proteasome-binding proteins are the valosin containing protein VCP (alias p97).

### 6.4. Protein Dislocase VCP Extracts Ubiquitinated Proteins from the Mitochondrial Membrane and Presents Them for Degradation

The vertebrate VCP (called Cdc48 in yeast), is a member of AAA-ATPase family involved in many cellular processes such as cell division, endoplasmic reticulum-associated protein quality control (ER-associated degradation/ERAD), and ubiquitin-dependent proteolysis. Recent studies also implicate VCP in regulating autophagy-mediated protein degradation via ubiquitin-dependent process. The VCP dislocase serves as the “motor” that mediates those cellular functions by binding to specific cofactors including Ufd1, Npl4, and p47. Those cofactors have ubiquitin-binding domains and interact with VCP as ubiquitin adaptors [120–122]. Based on the general model of VCP function within UPS, the target substrate is ubiquitinated by E1-E2-E3 enzyme cascade and VCP engages the polyubiquitin tail on the substrate protein through the above cofactors. The VCP then uses the energy from ATP hydrolysis to extract the ubiquitinated protein by separating it from its biding partners on the organelle membrane, and presenting it to the 26S proteasome for recycling [122]. Alternatively, the VCP-dislocated proteins can be delivered for autophagic protein degradation by VCP-mediated autophagosome. The importance of VCP for autophagy is confirmed by the inclusion body/familial VCP myopathy associated with frontotemporal dementia and Paget's disease of bone, caused by mutations in VCP [123]. The loss of VCP activity impairs maturation of ubiquitin-containing autophagosomes, which consequently fail to promote autophagy [124, 125].

The specific role of VCP during somatic cell mitophagy is to extract ubiquitinated outer mitochondrial membrane (OMM) proteins to the cytosol, for proteasomal degradation. The degradation of OMM-associated proteins, such as Mfn1 and Mcl2, is mediated by 26S proteasome in VCP-dependent manner [126, 127]. The OMM contains or can accommodate several E3 ubiquitin ligases. Specifically, Parkin, an E3 ubiquitin-ligase, is recruited to mitochondria and participates in mitophagy [127–129]. The mutation of Parkin-encoding* Park2* gene causes Parkinson's disease, which is associated with mitochondrial defects. Parkin mediates the elimination of the defective mitochondria by autophagosomes [129]. In addition, Parkin initiates the ubiquitination of OMM proteins, MFN1 and MFN2, thereby inducing both proteasome-dependent degradation and VCP-dependent mitophagy [129, 130]. Interestingly,* Drosophila* males lacking the Parkin gene are sterile and display mitochondrial pathology associated with the failure of spermatid individualization late in spermatogenesis [131]. Altogether, Parkin-mediated ubiquitination and VCP-induced degradation of ubiquitinated proteins can promote mitochondrial degradation by a dual route involving autophagy/mitophagy and proteasome-dependent degradation.

## 7. Conclusions

The mitochondria exist in almost all eukaryotic cells and are important for cellular energy production, calcium signaling, apoptosis, and many other cellular functions. Maternal inheritance of mitochondria and their DNA is universally observed in humans and most animals. The mutation and/or transmission of paternal mitochondrial genome are associated with various human diseases. The elimination of paternal mitochondria shortly after fertilization is the first line of defense to prevent potentially dangerous mitochondrial-genomic dysfunction. Reviewing current literature on mitochondrial inheritance, the elimination of paternal mtDNA can be accomplished by multiple mechanisms. Ubiquitination of germ cell mitochondria is observed during mammalian spermatogenesis and also detected after fertilization. The ubiquitinated sperm mitochondria typically disappear from early stage preimplantation embryos, while the exact timing of sperm mitophagy appears to vary among mammalian species. Embryo treatments with proteasomal and lysosomal inhibitors indicate the existence of a two-way mechanism of sperm mitochondrion degradation involving both proteasomal and lysosomal proteolysis, the latter being an autophagy/mitophagy endpoint.

**Figure 2:**
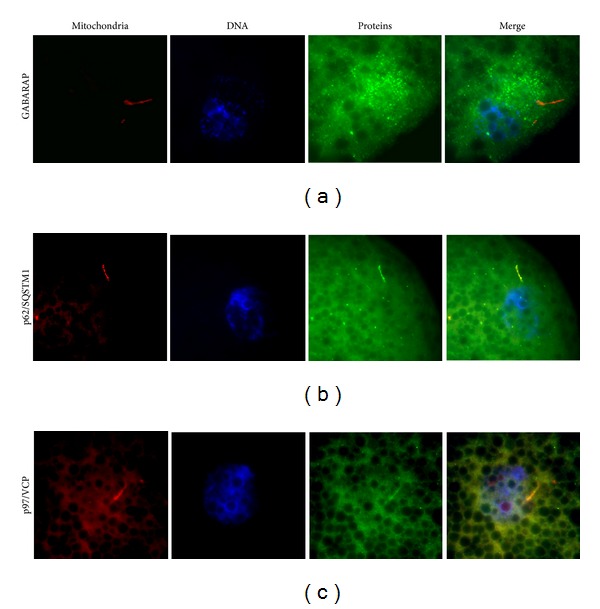
Immunofluorescence localization of GABARAP, p62/SQSTM1, and VCP in porcine zygotes. (a) Autophagy receptor/ubiquitin-like protein GABARAP accumulates around male pronucleus and sperm mitochondria of an embryo treated with proteasomal inhibitor MG132 (100 uM) at 30 hr after insemination. Inhibition of proteasomal proteolysis is known to induce compensatory activation of autophagic pathway. (b) Ubiquitin-binding protein p62/SQSTM1 is detected in the mitochondria region of spermatozoa in embryo cultured for 30 hr after IVF. SQSTM1 is an ubiquitin-receptor that links UPS to autophagic pathway. (c) Protein dislocase p97/VCP is present in the mitochondrial sheath of spermatozoa in a porcine zygote cultured for 30 hr after IVF. Dislocase VCP recognizes and extracts ubiquitinated mitochondrial membrane proteins, presenting them to 26S proteasome for degradation.

The recent findings in* C. elegans* help explain how ubiquitin-proteasome system and autophagy cooperate during the degradation of paternal mitochondria and mtDNA by the early embryo. Autophagy-related ubiquitin-receptors are detected in the paternal mitochondria inside the fertilized oocyte. It is thus possible that specific autophagy-related ubiquitin-binding proteins such as GABRAP, LC3, HDAC6, VCP, and SQSTM1 promote uniparental inheritance of mitochondria in mammals, as suggested by our preliminary data (Figure 2). Most likely, the interplay between proteasome-dependent degradation and sperm mitophagy exists in mammals and other taxa. Work is in progress to identify the mechanistic links between UPS and sperm mitophagy, which could impact the development of new treatments for human mitochondrial disease and infertility.

## Figures and Tables

**Table 1:** Nomenclature and aliases of autophagy-related genes.

Full name (human)	Yeast	*C. elegans *	Human/mammalian	Aliases	Detectable in spermatozoa	Function
Autophagy-related 5	N/A	Atg5	ATG5	ASP; APG5; APG5L; hAPG5; APG5-like	No	Early stage of autophagosome formation
GABA(A) receptor-associated protein	ATG8	Lgg-1	GABARAP	MM46; ATG8A; GABARAP-a	Yes	Nonselective sequestration of cytoplasmic material for vacuolar degradation
Microtubule-associated protein 1 light chain alpha	N/A	Lgg-2	MAP1LC3A	LC3; LC3 A; ATG8E; MAP1ALC3B	Yes	Recruits protein cargo to the phagophore/isolation membrane; remains associated with the mature autophagosome
Sequestosome 1	N/A	N/A	SQSTM1	p62; p60; A170	Yes	Binds to ubiquitinated proteins; interacts with both LC3 and GABARAP
Valosin containing protein/protein dislocase	CDC48	CDC-48.1	VCP	p97	Yes	Extracts ubiquitinated proteins from organelle membranes; presents them to 26S proteasome
Histone deacetylase 6	N/A	HAD-6	HDAC6	HD6	Yes	Transports ubiquitinated misfolded protein aggregates/aggresomes to phagophore
Parkin (PARK2)	N/A	pdr-1	PARK2	PDJ; PRKN	Not known	Ubiquitin ligase; induces selective autophagy of damaged mitochondria
